# Sex-specific hypothalamic neuropathology and glucose metabolism in an amyloidosis transgenic mouse model of Alzheimer’s disease

**DOI:** 10.1186/s13578-024-01295-5

**Published:** 2024-09-13

**Authors:** Guibo Qi, Han Tang, Pifang Gong, Yitong Liu, Chenzhao He, Jianian Hu, Siying Kang, Liang Chen, Song Qin

**Affiliations:** 1 Department of Anatomy, Histology and Embryology, School of Basic Medical Sciences, Fudan University, Shanghai, 200032 China; 2 State Key Laboratory of Medical Neurobiology and MOE Frontiers Center for Brain Science, Fudan University, Shanghai, 200032 China

**Keywords:** Alzheimer’s disease, Amyloid plaque, Glucose metabolism, Gliosis, Hypothalamus, Neuropathology

## Abstract

**Background:**

Amyloid toxicity and glucose metabolic disorders are key pathological features during the progression of Alzheimer’s disease (AD). While the hypothalamus plays a crucial role in regulating systemic energy balance, the distribution of amyloid plaques in the preoptic, anterior, tuberal, and mammillary regions of the hypothalamus in AD mice, particularly across both sexes, remains largely unclear. Our ongoing research aims to explore hypothalamic neuropathology and glucose metabolic disturbances in a well-described APP/PS1 mouse model of AD.

**Results:**

Immunocytochemical staining revealed that Old-AD-Female mice exhibited a greater hypothalamic Amyloid β (Aβ) burden than their Old-AD-Male counterparts, with the mammillary bodies showing the most severe accumulation. Analysis of ionized calcium binding adaptor molecule 1 (IBA1) immunoreactivity and *Iba1* mRNA indicated differential microgliosis based on sex, while tanycytic territory and ZO-1 tight junction protein expression remained stable in AD mice. Moreover, sex-specific peripheral glucose metabolic parameters (random and fasting blood glucose) seemed to be exacerbated by age. Old AD mice of both sexes exhibited limited hypothalamic activation (c-Fos + cells) in response to blood glucose fluctuations. Hypothalamic *Glut 1* expression decreased in young but increased in old female AD mice compared with age-matched male AD mice. Pearson correlation analysis further supported a negative correlation between hypothalamic Aβ load and random blood glucose in old AD groups of both genders, shedding light on the mechanisms underlying this amyloidosis mouse model.

**Conclusion:**

Aged APP/PS1 mice exhibit sex-specific hypothalamic neuropathology and differential glucose metabolism, highlighting distinct pathological mechanisms within each gender.

**Supplementary Information:**

The online version contains supplementary material available at 10.1186/s13578-024-01295-5.

## Introduction

Alzheimer’s disease (AD), an aging-related neurodegenerative disorder, is clinically characterized by memory loss and cognitive impairment [1, 2]. Its major hallmarks include amyloid β (Aβ) deposits and neurofibrillary tangles, leading to neuronal dysfunction and reactive gliosis [3–5]. Aβ plaques typically originate from pyramid neurons in the cortex and spread to other brain regions [6]. The hypothalamus, situated at the crossroads of peripheral, environmental, and neural inputs, integrates sensory information to regulate various physiological functions and behaviors [7]. Despite its complex structure comprising numerous neuron categories and nuclei, how each of the hypothalamic regions—preoptic, anterior, tuberal, and mammillary—experiences Aβ deposition remains largely unknown. Epidemiologically, approximately two-thirds of AD patients are women, indicating potential sex-specific differences in disease risk factors [8]. However, investigations into the sex-specific neuropathological variations in both human AD patients and amyloidosis transgenic mouse models remain limited.

Accumulating evidence suggests the role of hypothalamic glial populations in the AD brain [9, 10]. Microglia, expressing ionized calcium binding adaptor molecule (IBA1), drive the accumulation of Aβ plaques and cognitive deficits through a glycolysis/H4K12la/PKM2 (pyruvate kinase M2) positive feedback loop in AD mice [11]. Astrocytes undergo functional transformation into reactive astrogliosis, with the astrocytic α7 nicotinic acetylcholine receptor (α7nAChR) emerging as a potential biomarker candidate [12]. To date, the morphological and functional changes these cells undergo in the AD hypothalamus remain unclear. Indeed, tanycytes are radial glial-like ependymal cells located in the ventral part of the third ventricle (3 V), exhibiting intrinsic heterogeneity with α1, α2, β1, and β2 subpopulations [13]. Physiologically, tanycytes lining the ventral 3 V are connected at their apices by functional tight junctions [14] and participate in the remodeling of blood–hypothalamus barrier by expressing vascular endothelial growth factor-A [15]. These cells contribute to maintain energy balance and fat metabolism by sensing signals such as leptin and insulin, as well as circulating nutrients like glucose [16–18]. Recent lineage tracing studies suggest that tanycytes contribute to tissue repair under neural injury and may possess tumorigenic potential [19], indicating their response to pathological stimulation.

Another distinct feature of AD is disturbances in glucose and energy metabolism [20, 21]. It has been proposed that metabolic changes specific to certain brain regions precede the development of amyloid pathology and cognitive decline, with the hypothalamus undergoing significant metabolic alterations [22]. However, the relationship between systemic glucose levels and amyloid accumulation in aged AD mice of both sexes remains poorly understood. Robinson LS et al. provided evidence for sex-dependent effects of AD pathology on energy and glucose regulation [23], potentially linked to inflammation in the hypothalamus. Other studies have also reported sex differences in hypothalamic responses to metabolic challenges [24]. High-fat diets resulted in the greatest weight gain, adiposity, and glucose intolerance in 3xTg-AD females, accompanied by a significant increase in hypothalamic expression of glial fibrillary acidic protein (GFAP) and interleukin-1β (IL-1β) [25]. Interestingly, this effect was not observed in AD males [25], indicating a sexual dimorphic response to metabolic challenges.

Genetically, Rasse, R and colleagues established a transgenic AD mouse model known as APP/PS1 mice, which develop cerebral amyloidosis starting at 6–8 weeks of age [26]. While we previously traced reactive astrogliosis in the cortex of this AD mouse model and identified transcriptional alterations [27], the presence of amyloidosis in the hypothalamus has not been explored, nor has the metabolic profile of this AD mouse model been examined in a sex-specific manner.

The aim of this study is to investigate Aβ deposits in different regions of the hypothalamus in thoroughly characterized AD mice, focusing on both early and late pathological stages. We conducted immunocytochemical staining, mRNA measurements, and metabolic analyses on both young and old male and female AD mice. Additionally, we explored the relationship between hypothalamic Aβ pathology and systemic glucose metabolism, offering further insights into this amyloidosis mouse model.

## Materials and methods

### Animal care

Wild-type (WT) C57BL/6 J mice were purchased from the Shanghai SLAC Laboratory (Shanghai, China). AD mice (Thy1–APPKM670/671Nl, Thy1–PS1 L166P) harbor both the amyloid precursor protein (APP) with the KM670/671NL (Swedish) mutation and presenilin 1 (PS1) with the L166P mutation on a C57BL/6 J genetic background [26]. The mice were housed in groups of 4–6 per cage, provided with ad libitum access to food and water, and maintained under a 12-h light/dark cycle. Both young (3–4 months old) and old (14–15 months old) male and female mice were included in this study. All animal procedures were conducted in compliance with the guidelines and ethical regulations established by the Animal Care and Use Committee of Shanghai Medical College of Fudan University.

### Brain tissue preparation

Mice were anesthetized with pentobarbital sodium and xylazine, then subjected to transcardial perfusion with 1X ice-cold phosphate-buffered saline (PBS), followed by perfusion with 4% paraformaldehyde solution in PBS. Subsequently, the brains were carefully dissected and postfixed in 4% paraformaldehyde for 24–48 h at 4 ℃. The fixed brains were then transferred to 30% sucrose solution in PBS until they sank. After sinking, the brains were coronally sectioned to a thickness of 40 μm using a cryostat (Leica Microsystems) and stored in an anti-freeze solution at − 20 ℃ until immunohistochemical analysis. To perform c-Fos analysis, mice received an intraperitoneal injection (i.p.) of glucose solution (2 g/kg body weight) 2 h before scarification following the methods above.

### Immunocytochemistry

Brain sections were washed with 1X PBS and permeabilized with 0.3% Triton X-100 for 30 min at room temperature. Subsequently, the brain sections were blocked with 5% bovine serum albumin for 1 h at 4 ℃. Following overnight incubation with primary antibodies at 4 ℃, sections were washed and then incubated for 2 h with corresponding Alexa Fluor-conjugated secondary antibodies. Finally, the nuclei in each section were counterstained with Hoechst 33,258 (1 μg/ml). Antibodies used were as follows: 6E10 (1:500, AB_2564765, BioLegend), Aβ1-42 (1:500, ab224275, Abcam), GFAP (1:800, G3893, Thermo Scientific), IBA1 (1:1000, 019-19741, Waco), HuCD (1:1000, A-21271, Thermo Scientific); Vimentin (1:1500, AB_11212377, Merck & Millipore), ZO-1 (1:500, AB_2533147, Invitrogen), c-Fos (1:250, AB_2632380, PhosphoSolutions). Alexa Fluor 488-, 555-, or 647-conjugated corresponding secondary antibodies were purchased from Jackson ImmunoResearch (West Grove, PA).

### Image acquisition and analysis

All brain sections (at least 3 slices per animal) were imaged directly using a fluorescent microscope (EVOS M700 Color Imaging Systems). Additionally, a Leica SP8 microscope (Leica F1300439) was employed to further validate the results. Laser and scanning settings for images within each experiment were kept consistent for comparison between groups. Semi-quantification analysis was conducted by a blinded researcher using ImageJ software (NIH, Bethesda, MD). For amyloid load and IBA1% area, the color threshold tool in ImageJ was adjusted to label immunoreactive signaling while excluding background staining within the region of interest (ROI). The big warp plugin in ImageJ was utilized to merge brain atlas and acquired images for the counting of HuCD+cells. Following precise division, automatic cell counting was performed in various brain regions, including the anterior commissure (AC), medial preoptic nucleus (MPO), lateral preoptic nucleus (LPO), paraventricular nucleus (PVN), anterior hypothalamic nucleus (AHC), suprachiasmatic nucleus (SCN), supraoptic nucleus (SON), lateral hypothalamus (LH), dorsal medial nucleus (DMH), ventral medial nucleus (VMH), arcuate nucleus (ARH), tuberal nucleus (TuN), posterior hypothalamic nucleus (PH), supramammillary nucleus (SuM), and mammillary bodies (MM). Quantification of c-Fos immunoreactive cells was performed in feed-regulating nuclei, including DMH, VMN, ARH, TuN, and LH. For Sholl analysis, z-stack confocal images were captured at a resolution of 1024 × 1024 pixels. Photomontages were created with a step of 1 μm using × 40 magnification (between 6 and 10 frames per image). The number of intersections was calculated, starting with a radius of 10 pixels and subsequent shells set at 5 pixels per step. For the ratio of tanycytic layer to the third ventricular surface, the length of the tanycytic layer was reported to the total length of the ventricle. The tanycytic layer was defined as the distance from the bottom of the 3 V to the last tanycyte detected in the dorsal 3 V. The third ventricular surface was defined as the distance from the bottom of the 3 V to the top, measured using Hoechst staining.

### Metabolic study and food intake measurement

Blood samples were collected from the tail vein. Random blood glucose (RBG) was measured repeatedly in all WT and AD mice across both young and old groups. Fasting blood glucose (FBG) measurement was performed after overnight fasting. RBG and FBG levels of each animal were the average result of at least three independent measurements. In intraperitoneal glucose tolerance test (IPGTT), each mouse received an i.p. injection of glucose (2 g/kg body weight) after overnight fasting, and blood glucose levels were measured 0, 15, 30, 60, 90, and 120 min after i.p. injection. Subcutaneous fat mass was measured to indicate the wet weight of abdominal fat pad. Before actual food intake and rebound feeding measurements, mice were acclimatized to food hoppers in the cage at least 14 days. Actual food intake was measured continuously for 6 h: the difference between the pre-weighed and the remaining food was reported to 6 h. Fasting for 16 h minimizes the effects of systemic compensation including liver glycogenolysis. After a 16-h overnight fasting, ad libitum food intake was measured 1 h and 2 h after the light cycle started at 7:00–7:30.

### RNA isolation and RT-qPCR

As previously reported [28], total RNA was extracted from isolated hypothalamus tissues using TRIzol reagent. Subsequently, total RNA, along with random primers, was utilized to synthesize cDNA using the SuperScript III kit (Takara). We employed the TB Green system (Takara) for real-time quantitative PCR (RT-qPCR) analysis, performed with a 96-well ABI thermocycler (Applied Biosystems). Melting curves of each well were examined to confirm the amplification of a single product. Relative gene expression levels were quantified using the cycle threshold (Ct) method. The following primers were used: *Tnf-a* (forward: GGTGCCTATGTCTCAGCCTCTT; reverse: GCCATAGAACTGATGAGAGGGAG); *Ikbkg* (forward: TCTTCGGAGTCAGAGGGAACAG; reverse: TCCTGGAGTTCTCCGAGCAATG); *Iba1* (forward: TCTGCCGTCCAAACTTGAAGCC; reverse: CTCTTCAGCTCTAGGTGGGTCT); *Gfap* (forward: CACCTACAGGAAATTGCTGGAGG; reverse: CCACGATGTTCCTCTTGAGGTG); *Glut1* (forward: GCTTCTCCAACTGGACCTCAAAC; reverse: ACGAGGAGCACCGTGAAGATGA); *Glut3* (forward: CCGCTTCTCATCTCCATTGTCC; reverse: CCTGCTCCAATCGTGGCATAGA); *Gapdh* (forward: CCTACCCCCAATGTATCCGTT; reverse: TAGCCCAGGATGCCCTTTAGT).

### Statistics

All statistical analyses were conducted using GraphPad Prism software (version 8.2, GraphPad Software, La Jolla, CA, USA). Prior to further comparison, normality and lognormality tests were performed to ensure data normality. Data are expressed as mean ± standard error of the mean (SEM). Data from two groups were analyzed with a two-tailed unpaired Student’s t-test. Data of multiple groups were analyzed with one-way analysis of variance (ANOVA) followed by Turkey’s multiple comparison post hoc test. Two-way ANOVA followed by Bonferroni’s multiple comparison post hoc test was used to compare genotypes and genders over different gender groups. Pearson correlation analysis was performed using RStudio software (version: 2023.12.0 + 369) and visualized using the corrplot package. Significance levels were denoted on the graphs as follows: *, 0.01 ≤ *P* < 0.05; **, 0.001 ≤ *P* < 0.01; ***, 0.0001 ≤ *P* < 0.001.

## Results

### Sex-specific amyloid deposition with subregional heterogeneity in the hypothalamus of AD mice

**Fig. 1:**
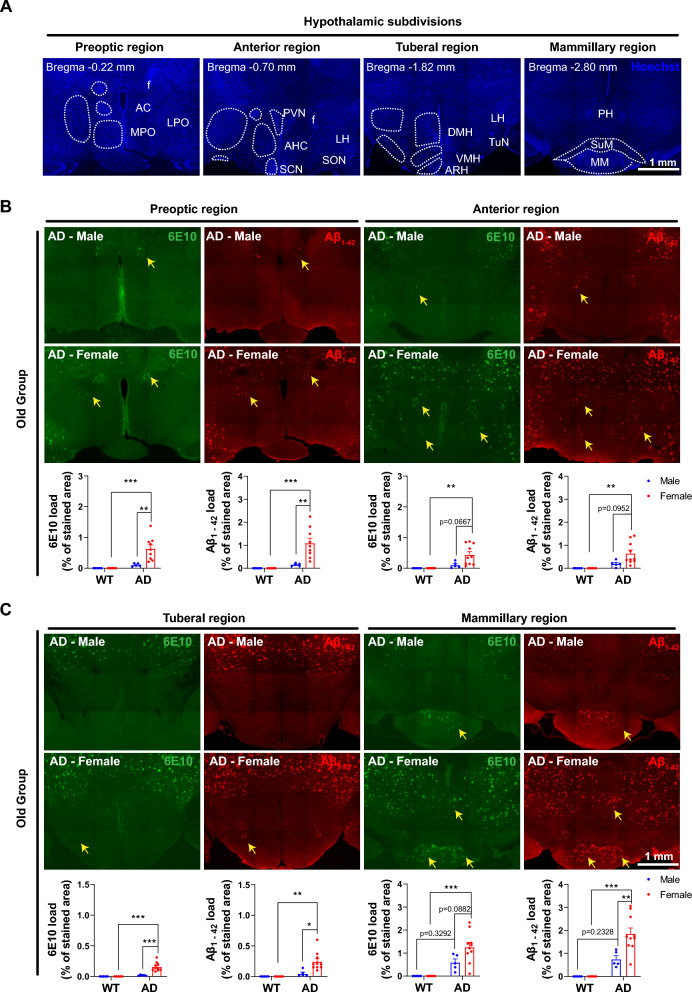
Comparative analysis of amyloid plaques in hypothalamic subdivisions. **A** Nucleus staining (Hoechst, blue) reveals four regions of hypothalamus in mice, including preoptic, anterior, tuberal, and mammillary regions. Nuclei indicated by dashed circles are labeled on the other side of the same slice. *AC* anterior commissure, *MPO* medial preoptic nucleus, *LPO* lateral preoptic nucleus, *PVN* paraventricular nucleus, *AHC* anterior hypothalamic nucleus, *SCN* suprachiasmatic nucleus, *SON* supraoptic nucleus, *LH* lateral hypothalamus, *DMH* dorsalmedial nucleus, *VMH* ventralmedial nucleus, *ARH* arcuate nucleus, *TuN* tuberal nucleus *PH* posterior hypothalamic nucleus, *SuM* supramammillary nucleus, *MM* mammillary bodies. Scale bar: 1 mm. **B** Representative images of 6E10 and Aβ_1–42_ co-staining in hypothalamic preoptic and anterior regions of Old-AD-Male and Old-AD-Female subsets. Corresponding quantification of 6E10 +and Aβ_1–42_ +load (n = 5–10). **C** Representative images of 6E10 and Aβ_1–42_ co-staining in hypothalamic tuberal and mammillary regions of Old-AD-Male and Old-AD-Female subsets. Corresponding quantification of 6E10 +and Aβ_1–42_ +load (n = 5–10). *p < 0.05; **p < 0.01; ***p < 0.001, two-way ANOVA followed by Bonferroni’s multiple comparison

To identify the Aβ burden in the hypothalamus, we initially segmented it into preoptic, anterior, tuberal, and mammillary regions, each comprising various nuclei (Fig. 1A). Subsequently, we performed co-staining of brain sections from Old-WT-Male, Old-WT-Female, Old-AD-Male, and Old-AD-Female mice using 6E10 and Aβ_1–42_ antibodies. Across all four regions, Aβ load was significantly higher in Old-AD-Female mice compared to Old-WT-Female mice (preoptic region: 6E10 p = 0.0008, Aβ_1–42_ p < 0.0007; anterior region: 6E10 p = 0.0086, Aβ_1–42_ p = 0.0070; tuberal region: 6E10 p < 0.0001, Aβ_1–42_ p = 0.0014; mammillary region: 6E10 p = 0.0004, Aβ_1–42_ p < 0.0001) (Fig. 1B, C). In contrast, we did not observe obvious amyloid plaques in the hypothalamus of Old-AD-Male mice, except in the mammillary region (6E10 load: p = 0.3292, Aβ_1–42_ load: p = 0.2328). Interestingly, Old-AD-Female mice exhibited significantly higher 6E10 +and Aβ_1–42_ +load compared to Old-AD-Male mice (preoptic region: 6E10 p = 0.0048, Aβ_1–42_ p = 0.0035; anterior region: 6E10 p = 0.0667, Aβ1–42 p = 0.0952; tuberal region: 6E10 p = 0.0001, Aβ_1–42_ p = 0.0100; mammillary region: 6E10 p = 0.0882, Aβ_1–42_ p = 0.0069) (Fig. 1B, C). To further confirm sex-specific amyloidosis, we applied confocal microscopy and detected more plaques in fornix and mammillary bodies of Old-AD-Female than Old-AD-Male mice (Fig. S1 A, B). However, no amyloid deposits were observed in the suprachiasmatic nucleus of the old groups (Fig. S1C).

Next, we examined whether the young group exhibited Aβ deposits in hypothalamic subdivisions. Images revealed that a few Aβ plaques were present in the mammillary region of both Young-AD-Male and Young-AD-Female mice (Fig. S2). Taken together, in this AD mouse line, the rostral-to-caudal hypothalamic microenvironment was differentially disrupted by senile plaques, with the mammillary region suffering from the most severe and earliest amyloid depositions. Old-AD-Female mice exhibited a heavier Aβ burden than their male counterparts, indicating sex-dependent amyloid pathology.

### Intact hypothalamic neuronal populations and NF-kb signaling pathways in AD mice

Sexual dimorphism of neuronal loss has been reported in the subiculum [29]. However, global neocortical neuron loss was not apparent until 8 months of age in the AD mouse model we used [26]. Given the presence of Aβ deposits along the rostral-to-caudal axis of the hypothalamus, it is intriguing to identify nucleus-specific neuronal populations. Therefore, we evaluated diverse hypothalamic nuclei (MPO, PVN, AHC, SCN, DMH, VMH, ARH, TuN, LH, PH, SuM, MM) in this AD mouse model using HuCD staining. However, no significant differences were observed in neuronal density among the 8 subsets, regardless of genotypes and genders (Fig. S3A, D). Functionally, we measured *Tnf-a* (a classic inflammatory factor) mRNA expression and found consistent levels of overall hypothalamic neuroinflammation (Old-AD-Male vs Old-WT-Male: p = 0.7009; Old-AD-Female vs Old-WT-Female: p = 0.1284) (Fig. S3B, E). Additionally, an inhibitor of nuclear factor kappa-B kinase subunit beta (*Ikbkβ*), was comparable among all subsets of the young and old group. Thus, neurons in various hypothalamic nuclei tend to remain intact, and the level of overall hypothalamic neuroinflammation is largely unaffected in both the early and late stages of this AD mouse line.

### Differential microgliosis based on sex in the MM of AD mice

**Fig. 2:**
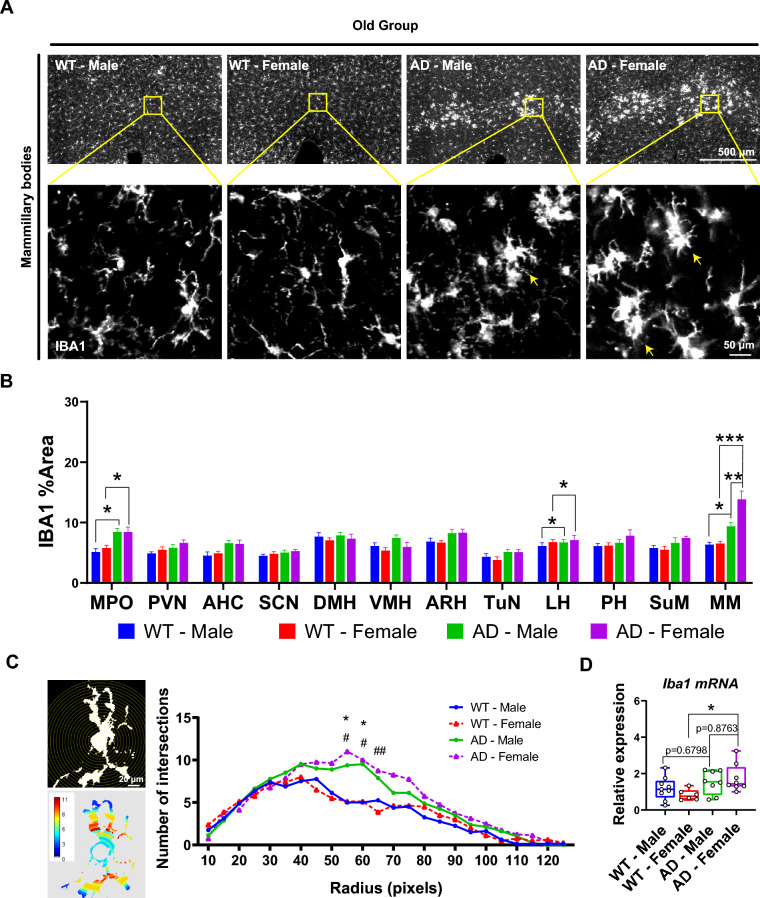
Microgliosis in various hypothalamic nuclei. **A** Representative image of IBA1 staining in mammillary bodies. Areas indicated by square are further amplified below via confocal. **B** Percentage of ROI occupied by IBA1-positive area (n = 6). **c** Binary picture of a single microglia against concentric circles and a heat map based on the same microglia. Number of intersections in Sholl analysis (n = 8). *: WT-male vs AD-male; ^#^: WT-female vs AD-female. *p < 0.05, ^#^p < 0.05, ^##^p < 0.01. **d** Relative mRNA expression of IBA1. *p < 0.05, two-way ANOVA followed by Bonferroni’s multiple comparison

Microglia, generally considered as a sensitive inflammatory predictor, have recently been supposed as AD culprits [30]. In the old AD groups, we observed significantly lager IBA1% area in the MPO (Old-AD-Male vs Old-WT-Male: p = 0.0133; Old-AD-Female vs Old-WT-Female: p = 0.0319), LH, (Old-AD-Male vs Old-WT-Male: p = 0.0491; Old-AD-Female vs Old-WT-Female: p = 0.0480) and MM (Old-AD-Male vs Old-WT-Male: p = 0.0450; Old-AD-Female vs Old-WT-Female: p < 0.0001) (Fig. 2A, B). In the MM, where Aβ plaques are produced robustly within the hypothalamus, microgliosis was more pronounced in Old-AD-Female mice than in Old-AD-Male subsets (p = 0.0023). Additionally, Sholl analysis revealed more interactions in both Old-AD-Male and Old-AD-Female mice compared to WT counterparts (Fig. 2C). Similarly, *Iba1* mRNA levels in the entire hypothalamus were consistent with protein expressions (Old-AD-Male vs Old-WT-Male: p = 0.6798; Old-AD-Female vs Old-WT-Female: p = 0.0482) (Fig. 2D). These results suggest differential microgliosis based on sex in aged AD mice.

In the young groups, a larger IBA1% area was only found in the MM (where Aβ plaques are produced earliest) of Young-AD-Female compared with Young-WT-Female (p = 0.0479) (Fig. S4A, B). No differences were observed in *Iba1* mRNA expression (Fig. S4C). Furthermore, we investigated astrocytic reaction in the MM to confirm gliosis. In the young group, GFAP antibody immunostaining were undetectable, and *Gfap* mRNA expression was similar among the 4 subsets (Fig. S4D, E). In contrast, both Old-AD-Male and Old-AD-Female mice displayed GFAP immunoreactivity in the MM (Fig. S4F). Additionally, *Gfap* mRNA was increased in Old-AD-Female compared to Old-AD-Male mice (p = 0.0167) (Fig. S4G).

### Stable tanycytic territory and honeycombed tight junction protein ZO-1 in AD mice

**Fig. 3:**
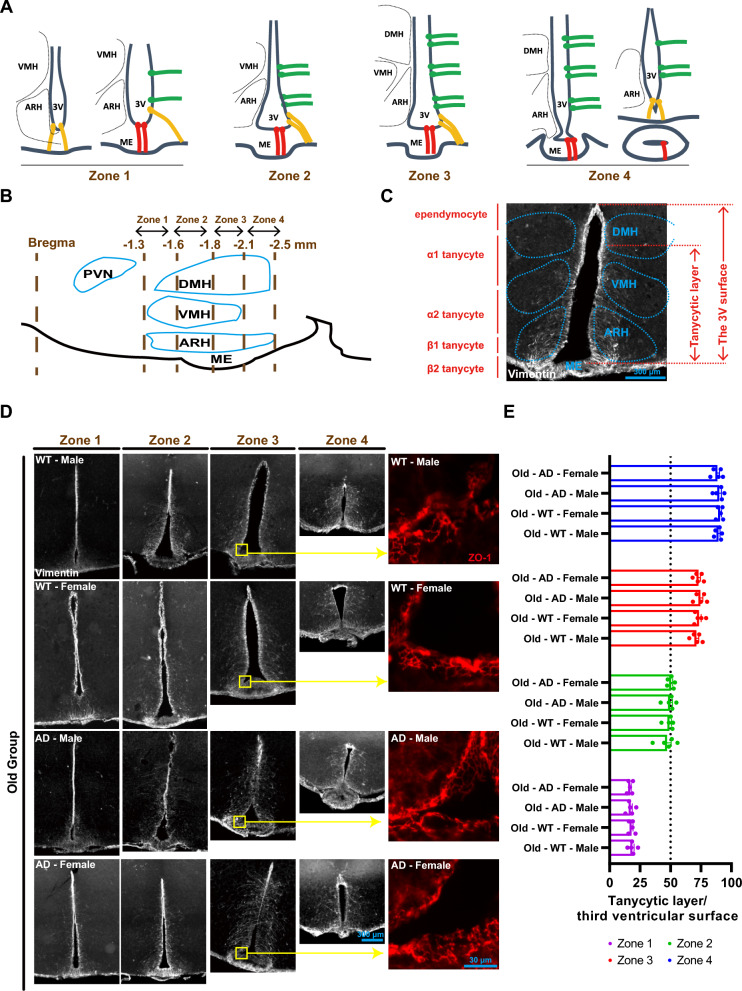
Tanycytes lining the lateral and ventral wall of the third ventricle (3 V). **A** Classical distribution of tanycytes in the tuberal region of the hypothalamus (adapted with the permission of Prof. Fanny Langlet). **B** The sagittal schematic showing zone 1 (from bregma – 1.3 to – 1.6 mm), zone2 (from bregma – 1.6 to – 1.8 mm), zone 3 (from bregma – 1.8 to – 2.1 mm) and zone 4 (from bregma – 2.1 to – 2.5 mm) along the anteroposterior axis. **C** Immunohistochemistry for the intermediate filament vimentin (white) showing all subtypes of tanycytes and ependymocytes populating the 3 V wall of mice. The tanycytic layer (distance from the 3 V bottom to the top tanycytes) and the 3 V surface (distance from the bottom to the top of 3 V) are delineated. Scale bar, 300 μm. **D** Vimentin-positive tanycytes distribute from zone 1 to zone 4 in the old group. Pattern of tight junction protein ZO-1 (red) is honeycombed in bottom 3 V of the old group. **E** The ratio of 3 V surface occupied by tanycytic layer revealed no differences in tanycyte territory between old groups of WT and AD mice (n = 5). Two-way ANOVA with Bonferroni’s post hoc test

There is growing evidence indicating that tanycytes play a role in maintaining brain glucose sensing [31]. Due to their peculiar distribution, tanycytes are conventionally divided into four subpopulations based on their dorsal–ventral position [32]. In our study, we initially divided the tuberal region of the hypothalamus (from bregma – 1.3 to – 2.5 mm) into four zones: zone 1 (from bregma – 1.3 to – 1.6 mm), zone 2 (from bregma – 1.6 to – 1.8 mm), zone 3 (from bregma – 1.8 to – 2.1 mm), and zone 4 (from bregma – 2.1 to – 2.5 mm) along the antero-posterior axis (Fig. 3A, B). Tanycytic territory was assessed by the ratio of the tanycytic layer to the surface of the 3 V vimentin-positive (vimentin+) tanycytes (Fig. 3C). Across zones 1 to 4, tanycytic territory remained consistent among the four subsets of the old group (Fig. 3D, E). We then examined tight junction proteins, particularly ZO-1, which are crucial for the structure of blood–cerebrospinal fluid barriers and prevent the diffusion of blood-borne molecules into the cerebrospinal fluid [33]. In zone 3 of Old-AD-Male and Old-AD-Female mice, tanycytes exhibited a continuous ZO-1 +honeycomb pattern at the apex and predominantly lined the 3 V wall adjacent to ME (Fig. 3D). Similarly, stable tanycytic territory and ZO-1 +honeycomb patterns were also observed in the young group (Fig. S5). These results suggest that tanycytic territory and ZO-1 tight junction proteins are not susceptible to AD pathology in this mouse model.

### Sex differences of systemic glucose metabolism in young and old AD mice

**Fig. 4:**
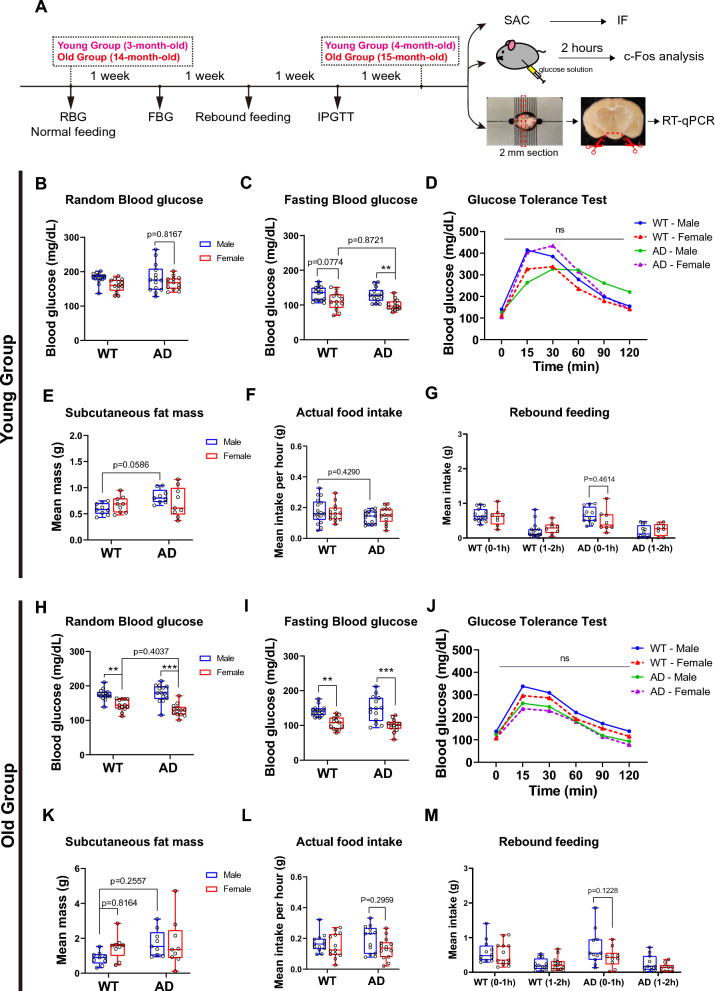
Systemic glucose metabolism and food intake of the young and old group. **A** The workflow displaying experimental timelines in this study. Metabolic measurements are 1 week apart from adjacent ones, allowing for animal recovery. Groups of both ages, genotypes and genders, totally 8 subsets (Young-WT-Male, Young-WT-Female, Young-AD-male, Young-AD-female, Old-WT-Male, Old-WT-Female, Old-AD-Male, Old-AD-Female), are sacrificed after 4 weeks. The schematic pictures also present processes before c-Fos analysis and RT-qPCR. Young groups: **B** Random blood glucose levels (n = 13–14). **C** 16-h Fasting blood glucose levels (n = 13–14). **D** IPGTT (n = 6). **E** Subcutaneous fat mass (n = 9). **F** Food intake during the day (n = 13–17). **G** Rebound food intake after 16-h fasting (n = 8–15). Old groups: **H** Random blood glucose levels (n = 13–14). **I** 16-h Fasting blood glucose levels (n = 13–14). **J** IPGTT (n = 6). **K** Subcutaneous fat mass (n = 9). **L** Food intake during the day (n = 13–14). **M** Rebound food intake after 16-h fasting (n = 10–13). **p < 0.01; ***p < 0.001, two-way ANOVA followed by Bonferroni’s multiple comparison

To explore the toxicity induced by cerebral Aβ plaques and its association with various peripheral and central abnormalities [34], we assessed multiple parameters of glucose metabolism in both young and old groups following the outlined workflow (Fig. 4A). In the young group, the value of FBG (a challenged glucose metabolic parameter) was significantly reduced in Young-AD-Female compared to Young-AD-Male mice (p = 0.0036), and showed a decreasing trend in Young-WT-Female compared to Young-WT-Male mice (p = 0.0774) (Fig. 4C). Intriguingly, the FBG of Young-AD-Female mice was similar to that of Young-WT-Female mice (p = 0.8721). Additionally, no significant differences were observed among the four young subsets in the values of RBG, GTT, subcutaneous fat mass, actual food intake, or rebound feeding (Fig. 4B, D, E, F, G). To sum up, the glucose metabolic profile of young AD mice differed significantly between the two genders.

In the old group, RBG (p < 0.0001) and FBG (p < 0.0001) were significantly lower in Old-AD-Female mice compared to Old-AD-Male mice (Fig. 4H, I). Unexpectedly, RBG (p = 0.0011) and FBG (p = 0.0020) were also significantly lower in Old-WT-Female compared to Old-WT-Male mice, while these statistical differences did not exist between Young-WT-Female and Young-WT-Male mice (Fig. 4B). In either Old-AD-Male or Old-AD-Female mice groups, both RBG and FBG are similar to WT control groups (Fig. 4G, H). Glucose tolerance and all feeding behavioral parameters of Old-AD-Female mice were comparable to those of Old-AD-Male mice (Fig. 4J, K, L, M). These findings suggest that the level of systemic blood glucose differs in a sex-specific manner, indicating that age-related glucose metabolism seems to be independent of AD genotype.

### Low activation of feed-regulating hypothalamic nucleus in aged AD mice

**Fig. 5:**
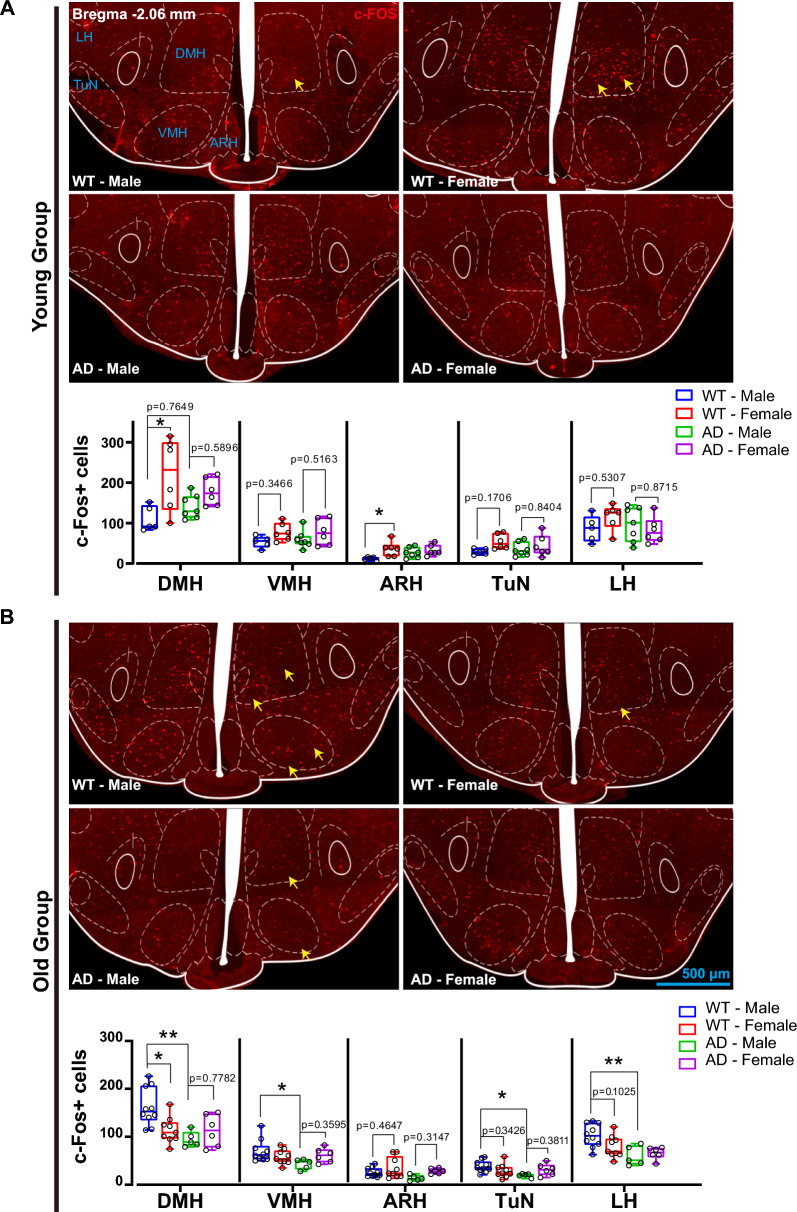
Glucose-induced cell activation of in hypothalamic metabolism-regulating nuclei of 8 subsets. **A** Images of c-Fos immunofluorescence in the young group (Young-WT-Male, Young-WT-Female, Young-AD-male, Young-AD-female). Nucleus-specific quantification of c-Fos + cells in DMH, VMH, ARH, TuN, and LH (n = 5–7). A total of 19,264 c-Fos + cells were counted. Scale bar, 500 μm. **B**. Images of c-Fos immunofluorescence in the old group (Old-WT-Male, Old-WT-Female, Old-AD-Male, Old-AD-Female). Nucleus-specific quantification of c-Fos + cells in DMH, VMH, ARH, TuN, and LH (n = 5–9). Scale bar, 500 μm. *p < 0.05; **p < 0.01, two-way ANOVA followed by Bonferroni’s multiple comparison

We next traced the central response to acute glycemia upregulation by measuring cellular c-Fos expression in hypothalamic nuclei involved in regulating feeding and glucose metabolism. Two hours after intraperitoneal injection of a glucose solution (2 g/kg), we found that the numbers of c-Fos+cells in the DMH, VMH, ARH, TuN, and LH were identical among the young groups (Young-WT-Male, Young-WT-Female, Young-AD-Male, Young-AD-Female mice) (Fig. 5A). Exceptionally, the number of c-Fos+cells in the DMH (p = 0.0130) and ARH (p = 0.0363) were slightly higher in Young-WT-Female mice compared to Young-WT-Male mice (Fig. 5A).

In the old group, we found significantly fewer c-Fos+cells in the DMH (p = 0.0035), VMH (p = 0.0490), TuN (p = 0.0390), and LH (p = 0.0058) of Old-AD-Male mice compared to Old-WT-Male mice (Fig. 5B). Similarly, a small number of c-Fos+cells were observed in Old-AD-Female mice, although they did not reach a significant level compared with WT controls (Fig. 5B). Additionally, c-Fos+cells in the DMH of Old-WT-Female mice were slightly decreased compared to Old-WT-Male mice (p = 0.0150) (Fig. 5B). Overall, both Old-AD-Male and Old-AD-Female mice exhibited limited hypothalamic activation in response to blood glucose fluctuations.

### Systemic glucose metabolism was negatively correlated with hypothalamic amyloid pathology in aged AD mice of both sexes

**Fig. 6:**
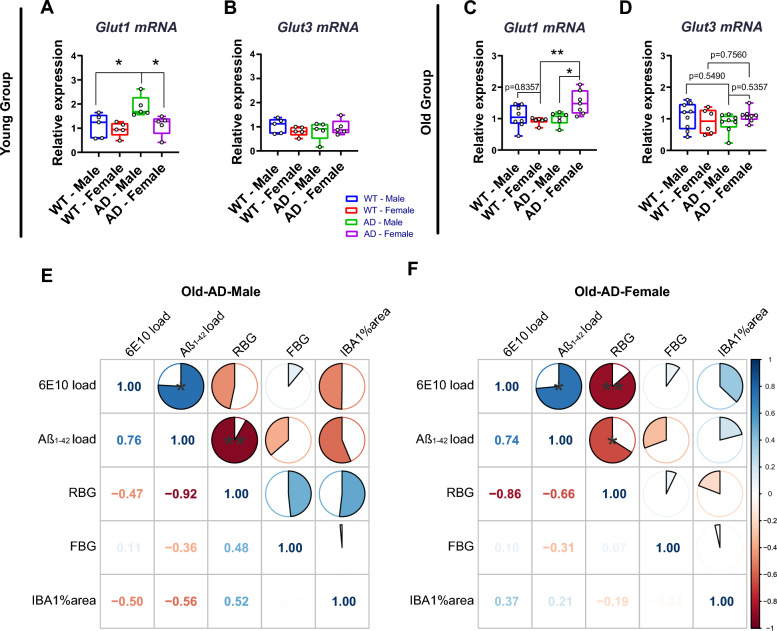
Relative mRNA expression of Glut1 (**A**, **B**), Glut3 (**C**, **D**) in the hypothalamus of both young and old male and female WT and AD groups mice. Pearson correlation of several parameters in Old-AD-Male and Old-AD-Female groups (E, F). The correlation coefficient was listed on the left and below side of each matrix. *p < 0.05; **p < 0.01

The glucose transporter transporter 1 (GLUT1) at the blood–brain barrier (BBB) mediates glucose transport into the brain, and GLUT1 deficiency exacerbates AD-related cerebrovascular degeneration in the cortex and hippocampus [35]. Neurons primarily obtain extracellular glucose as fuel, mainly relying on glucose transporter 3 (GLUT3) [36]. We observed slightly increased hypothalamic *Glut1* mRNA levels in Young-AD-Male compared to either Young-WT-Male (p = 0.0397) or Young-AD-Female mice (p = 0.0470) (Fig. 6A). *Glut3* mRNA expression was similar among the four subsets of the young group (Fig. 6B). Surprisingly, in the old group, hypothalamic *Glut1* mRNA levels were significantly higher in Old-AD-Female compared to either Old-WT-Female (p = 0.0089) or Old-AD-Male mice (p = 0.0269). There was no statistical difference in *Glut3* mRNA expression between Old-AD-Male and Old-AD-Female (p = 0.5357).

Finally, we investigated whether hypothalamic amyloid pathology was associated with systemic glucose metabolic parameters in Old-AD-Male and Old-AD-Female mice. Pearson analysis showed that in the hypothalamus of Old-AD-Male mice, Aβ_1–42_ load was negatively correlated with RBG (r = − 0.93, p < 0.01). Both 6E10 load (r = − 0.86, p < 0.01) and Aβ_1–42_ load (r = − 0.66, p < 0.05) in Old-AD-Female mice were strongly and negatively correlated with RBG. Additionally, the percentage of IBA1-covered areas in the total hypothalamus of Old-AD-Male and Old-AD-Female mice was not statistically associated with Aβ deposition (Aβ_1–42_ load or 6E10 load).

## Discussion

This study reveals an uneven distribution of senile plaques in the rostral-to-caudal hypothalamus of AD mice, with the earliest and densest accumulation observed in the MM region. Old-AD-Female mice exhibited a higher Aβ burden and gliosis compared to Old-AD-Male mice. In contrast, tanycytic territories remained stable, and the honeycombed patterns of ZO-1 were retained in the old AD mice group. Sex-specific peripheral glucose metabolic parameters (RBG and FBG) tend to be exacerbated by age, and hypothalamic cellular activation in old AD mice is limited in response to blood glucose fluctuations. Additionally, hypothalamic Aβ load was negatively associated with RBG in both Old-AD-Male and Old-AD-Female mice.

Extracellular deposition of Aβ is a primary neuropathologic hallmark in AD [37]. While Aβ plaques in the cortex and hippocampus researchers have been extensively investigated in AD mouse models, evaluation of hypothalamic amyloidosis is comparatively limited. In a study by Carrero et al. [38], no Aβ immunostaining was detected in the hypothalamus of 12-month-old male APP/PS1 mice; however, confocal imaging revealed intracellular Aβ immunostaining within hypothalamic neurons. Similarly, Tsui KC and colleagues provided a panoramic view of Aβ distribution in 9-month-old female 5xFAD mice, claiming no amyloidosis in specific hypothalamic regions such as the PVN, SCN, LH, and TuN. In their study, however, Fig. 6h showed mild Aβ deposition in the lateral and medial mammillary nucleus, despite the lack of detailed results description [39]. In line with this, our observations revealed abundant 6E10 +and Aβ_1–42_ plaques in the MM of 15-month-old APP/PS1 mice. Additionally, Aβ deposition in the preoptic, anterior, and tuberal region of the hypothalamus was mild in Old-AD-Female mice and minimal in Old-AD-Male mice. Among these regions, we observed that the fornix, a component of the limbic system, was particularly affected by Aβ deposition. Clinically, the fornix has been identified as a potential therapeutic target of AD to enhance inter-regional connectivity and improve memory function, impairment of which may stem from Aβ deposition [40, 41].

Studies exploring the effect of sex on AD appear to be contradictory due to variations in the ages and construction methods of AD mice utilized. For instance, a study based on non-transgenic amyloidosis mice model (involving intracerebroventricular injection of Aβ_1–42_) found similar alterations in long-term potentiation (LTP) and long-term depression (LTD) in both male and female groups [42]. One possible explanation is that mice used were 3–6 months old, and any sexual differences may have been too subtle to detect. Sexual dimorphism may manifest in later stages of AD [43]. In contrast, another study described more prominent amyloid plaques in the hippocampus of female 3xTg mice compared to male 3xTg mice, with estrogen deficiency-induced PKA-CREB-MAPK (protein kinase A-cAMP response element-binding protein and p38–mitogen-activated protein kinases) signaling disorder involved [44]. Similarly, we observed higher Aβ loads of 6E10 +and Aβ1-42 +in the hypothalamus of old AD mice, with female developing more plaques than male subsets. Sex-dependent hypothalamic neuropathology might reflect distinct compensatory mechanisms during the late stages of AD pathogenesis. In contrast, during the early stages of AD, genotype rather than sex appears to be the dominant factor in hypothalamic Aβ deposition, as evidenced by the presence of a few Aβ plaques in the mammillary region of both young male and female AD mice. However, our study does not specifically investigate the key molecular players or signaling pathways underlying sex differences in hypothalamic neuropathology.

Post-mortem examination of elderly AD patients has revealed neuronal loss in the SCN [45], LH [46] and MM [47], as well as the presence of dystrophic axons and abnormal spines in the SON and PVN [48]. Some AD transgenic mouse models replicated the neuronal loss observed in human patients. Poon CH [29] reported a significant decrease in the number of NeuN+cells in the subiculum of 6-month-old male and female 5xFAD mouse model. Trujillo-Estrada [49] reported reduced somatostatin+neurons in the subiculum of 4-month-old male AβPP751SwedLondon/PS1M146L mice. In our study, to objectively assess neuronal populations, we evaluated neuronal density per unit area rather than total neuronal numbers in various hypothalamic nuclei. Consequently, the density of HuCD+neurons was minimally affected by Aβ cytotoxicity in this AD mouse line. Consistent with normal density, stable *Glut 3* mRNA in the AD hypothalamus indicated that the neural capacity for transporting glucose was probably retained. However, a limitation of our study is that we did not quantify functional neuronal subgroups in the hypothalamus of AD mice via fluorescence-activated cell sorting (FACS). In addition, faulty autolysosome acidification in neurons of AD mouse models leads to intracellular autophagic accumulation of Aβ peptides [50]. Therefore, it would be interesting to explore early functional changes in neuronal lysosomes in the hypothalamus of APP/PS1 mice.

Glial activation is a complex neuropathology in AD [51]. As a recognized cellular marker of neuroinflammation, microgliosis is predominantly localized to the core of Aβ deposits [52]. Although *Tnf-a* and *Ikbkβ* mRNA levels in our study indicate no significantly elevated neuroinflammation in the overall AD hypothalamus, we consider it to be an integrated consequence of hypothalamus-residing cells (including neurons, microglia, astrocytes, and others). The percentage of IBA1-positive area in the MM was greater in Old-AD-Female mice than in Old-AD-Male mice, corresponding to the 6E10 +and Aβ1–42 +amyloid load. Since severe microgliosis was detected in the LH and MM but not in other hypothalamic nuclei, such localized inflammation might be too subtle to detect. In Pearson correlation analyses of aged APP/PS1 mice, we found no significant correlations between hypothalamic IBA1% areas and Aβ deposition. One potential reason is that, to remain consistent with the measurement standard of Aβ load, we analyzed IBA1-covered areas of the total hypothalamus (beyond MM). Another reason could be the presence of complicated microglial subgroups in various hypothalamic nuclei, which may exhibit different IBA1 immunoreactivity. Tools targeting proinflammatory and anti-inflammatory microglial subtypes with high specificity may help interpret our findings. GLUT1 predominantly facilitates glucose uptake in microglia [53] and mediates microglial proinflammatory activation [54]. In our study, *Glut 1* mRNA expression was decreased in Young-AD-Female mice and increased in Old-AD-Female mice, implying variable microglial glucose metabolism in the AD hypothalamus.

Tanycytes have been speculated to be the missing link between type 2 diabetes and AD and are therefore considered for targeted gene-editing and stem cell–based patient-specific therapies for AD [55, 56]. To the best of our knowledge, our study is the first to dissect tanycytes in a mouse model of AD. We observed no atrophic tanycytic territory and honeycombed ZO-1 pattern in AD mice, implying a normal blood–cerebrospinal fluid barrier (BCSFB) constituted by the tanycyte population. Due to technical limitations, we did not reveal ultrastructural connections with organelles in tanycytes, such as mitochondria and lysosomes [57]. Functionally, hypothalamus-controlled downstream metabolic effects are partially mediated by tanycytes [58, 59]. Activation of tanycytes through the arcuate neuronal network leads to acute hyperphagia [60], and they are proven essential for rodents to initiate a meal after fasting [61]. With high functional heterogeneous [62], a subpopulation of tanycytes expresses key taste transduction genes and regulate glucose homeostasis [63]. Thus, it is highly possible that tanycytes in AD mice retain the ability to sustain normal food intake, unaffected by age and sex.

Hypothalamic-based metabolic disorders are another critical pathological feature in AD [64, 65], often preceding amyloid plaques [66, 67]. Negative energy balance (decreased body weight, food intake, and energy expenditure) and metabolic dysfunctions (insulin, leptin, ghrelin) have been reported in 6-month-old male and female 5xFAD mice [68]. In our study, however, no significant difference was found in feeding behaviors of all animal groups, suggesting negligible impact from sex, age, and AD pathology. Expression of c-Fos reveals a limited response of AD hypothalamic feed-related nuclei when challenged with raised blood glucose. Notably, glucose-induced c-Fos + cells were more dramatically reduced in Old-AD-Male compared to Old-AD-Female mice, displaying sex-dependent patterns. We infer that in Old-AD-Male mice, hypothalamic neurons transition from activated states to resting states to adapt to rapidly rising glycemia. In contrast, neurons in Old-AD-Female mice fail to complete this transition, likely due to higher Aβ deposition. In line with our results, other studies have reported that alterations in hypothalamic glucose sensing and utilization are mild in 2–3-month-old APP+mice [69], and 11-month-old male APP/PS1 mice fed standard chow showed normal FBG and glucose tolerance compared with WT mice [70].

Additionally, we showed significantly different RBG and FBG in aged AD male and female mice. Unexpectedly, such sex-based difference also existed in Old-WT-Male and Old-WT-Female mice (but not in Young-Female-WT and Young-Male-WT mice). Other studies have reported that 14-month-old male C57BL/6 mice had higher baseline blood glucose than female following glucose administration [71], suggesting sex-specific impairments in blood glucose regulation [72]. The underlying reason might be that age combined with sex (or age interacting with sex) has a greater impact than sex or AD genotype alone, causing a statistical difference in glucose metabolic parameters in old WT groups. By contrast, FBG showed a decreasing trend in Young-WT-Female compared with Young-WT-Male mice (and statistically differed between Young-AD-Female and Young-Male-AD mice), and RGB was comparable across all four young subsets, further emphasizing the significant influence of age on metabolism. We thus infer that sex differences in systemic glycemia seem to be exacerbated by age but are unlikely to be influenced by AD status. Furthermore, Pearson correlation analysis revealed that hypothalamic Aβ load was negatively correlated with RGB in both old AD male and female mice. In young AD male and female mice, a few Aβ plaques appeared in the mammillary region of the hypothalamus, implying a potential link between blood glucose metabolism and hypothalamic AD pathology. Notably, more studies are warranted to confirm this link in the early pathological stages.

Limitations of this study also include the lack of soluble Aβ examination in the blood sample from both male and female AD mice. Soluble Aβ proteins are promising early diagnostic biomarkers of AD, as their imbalance in production and clearance across the blood–brain barrier has been implicated [73]. Moreover, we did not directly measure glucose levels in the interstitial fluid of the hypothalamic parenchyma and compare them with peripheral glucose levels. It would be fascinating to clarify whether the AD hypothalamus fails in glucose sensation, and transportation, or rather experiences a decline in energy demand. We primarily explored blood glucose levels in our metabolic study and did not investigate other metabolic parameters, such as insulin, leptin, ghrelin, which limits our findings from being generalized to integrated metabolic disturbances. Functionally, resting-state functional connectivity of the hypothalamic precuneus/posterior cingulate cortex were diminished in AD patients [74]. This suggests that in addition to hypothalamic histopathology changes, signaling exchanges between the hypothalamus and other brain regions may also be impaired in AD. Investigating key and vulnerable monosynaptic projections from other brain regions to the integrative hypothalamus, which mediate glucose disorders, would be of great interest in the future research.

## Conclusions

**Fig. 7:**
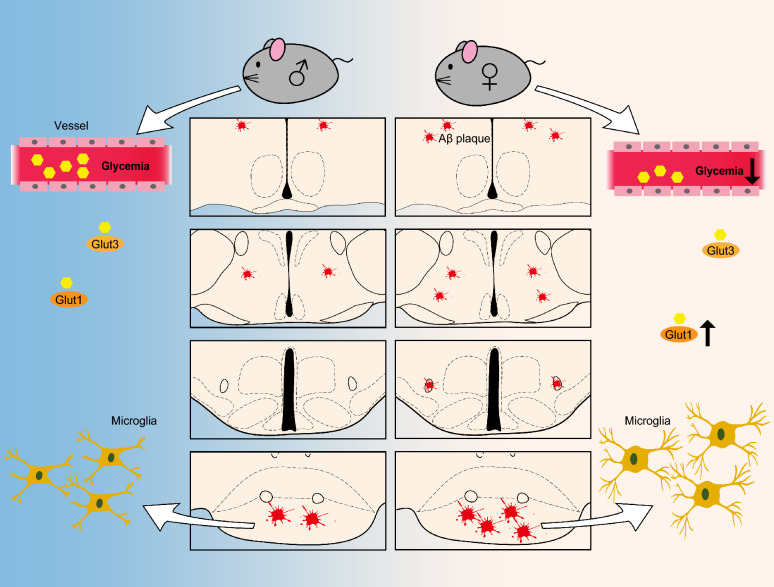
Schematic diagram showing sexual differences in the AD mice model. The pictures reflected lower glycemia in Old-AD-Female mice, coupled with heavier Aβ burden, elevated *Glut1* expression and activated microglia

In summary (Fig. 7), we have demonstrated more extensive hypothalamic amyloid plaques in females than males in the late stage of this AD mouse model. The MM exhibits the earliest and heaviest Aβ load within the hypothalamus, accompanied by microgliosis. Sex-specific systemic glucose metabolism (RBG and FBG) seems to be exacerbated by age, and c-Fos expression in hypothalamic nuclei regulating feeding is limited in the old AD groups of both sexes, indicating impaired glucose metabolic adaptation. These findings underscore the importance of considering sex-specific hypothalamic amyloidosis and age-related sex differences in blood glucose metabolism.

## Supplementary Information

Additional file 1: Fig. S1 Representative confocal images of amyloid plaques in the Old-AD-Male and Old-AD-Female subsets. 6E10+ and Aβ1–42+ plaques are observed in the fornix (A), mammillary bodies (B), but not in the suprachiasmatic nucleus. Scale bar, 50 μm. Fig. S2 Analysis of amyloid plaques in hypothalamic subdivisions of the young group. Representative images of 6E10 and Aβ1–42 co-staining in hypothalamic preoptic, anterior (A), tuberal, and mammillary (B) regions of Young-AD-Male and Young-AD-Female subsets. Fig. S3 Neurons in various hypothalamic nuclei and neuroinflammation elements. A Images of HuCD immunofluorescence in the young group. Scale bar, 200 μm. Quantification of the number of HuCD+ neurons in various nuclei. B, C Tnf-a and Ikbkβ mRNA expression in the young group were evaluated. D Images of HuCD immunofluorescence in the young group. Scale bar, 200 μm. Quantification of the number of HuCD+ neurons in various nuclei. E, F Tnf-a and Ikbkβ mRNA expression in the young group were evaluated. Fig. S4 Staining of IBA1 and GFAP in various hypothalamic nuclei. A Representative image of IBA1 staining in mammillary bodies of the young group. B Percentage of ROI occupied by IBA1-positive area (n = 3). Representative image of GFAP staining in mammillary bodies of the young group (c) and old group(d). *p < 0.05, two-way ANOVA followed by Bonferroni’s multiple comparison. Fig. S5 Vimentin-positive tanycytes distribute from zone 1 to zone 4 in the young group. Pattern of tight junction protein ZO-1 (red) is honeycombed in bottom 3V of the young group

## Data Availability

The datasets used and/or analyzed during the current study are available from the corresponding author upon reasonable request.

## Abbreviations

AD: Alzheimer’s disease
Aβ: Amyloid beta
α7nAChR: α7 nicotinic acetylcholine receptor
3 V: Third ventricle
WT: Wild-type
APP: Amyloid precursor protein
PS1: Presenilin 1
PBS: Phosphate-buffered saline
i.p.: Intraperitoneal injection
ROI: Region of interest
AC: Anterior commissure
MPO: Medial preoptic nucleus
LPO: Lateral preoptic nucleus
PVN: Paraventricular nucleus
AHC: Anterior hypothalamic nucleus
SCN: Suprachiasmatic nucleus
SON: Supraoptic nucleus
LH: Lateral hypothalamus
DMH: Dorsal medial nucleus
VMH: Ventral medial nucleus
ARH: Arcuate nucleus
TuN: Tuberal nucleus
PH: Posterior hypothalamic nucleus
PKM2: Pyruvate kinase M2
GFAP: Glial fibrillary acidic protein
IL-1β: Interleukin-1B
SuM: Supramammillary nucleus
MM: Mammillary bodies
RBG: Random blood glucose
FBG: Fasting blood glucose
IPGTT: Intraperitoneal glucose tolerance test
RT-qPCR: Real-time quantitative PCR
Ct: Cycle threshold
SEM: Standard error of the mean
ANOVA: Analysis of variance
*Ikbkβ*: An inhibitor of nuclear factor kappa-B kinase subunit beta
BBB: Blood–brain barrier
GLUT1: Glucose transporter 1
GLUT3: Glucose transporter 3
LTP: Long-term potentiation
LTD: Long-term depression
PKA-CREB-MAPK: Protein kinase A-cAMP response element-binding protein and p38–mitogen-activated protein kinases
FACS: Fluorescence-activated cell sorting
BCSFB: Blood–cerebrospinal fluid barrier
