# Supplementary figures and images for: Sex-specific hypothalamic neuropathology and glucose metabolism in an amyloidosis transgenic mouse model of Alzheimer’s disease
Source: Cell Biosci. 2024 Sep 13;14:120. doi: 10.1186/s13578-024-01295-5 (PMC11395863; doi:10.1186/s13578-024-01295-5)

A

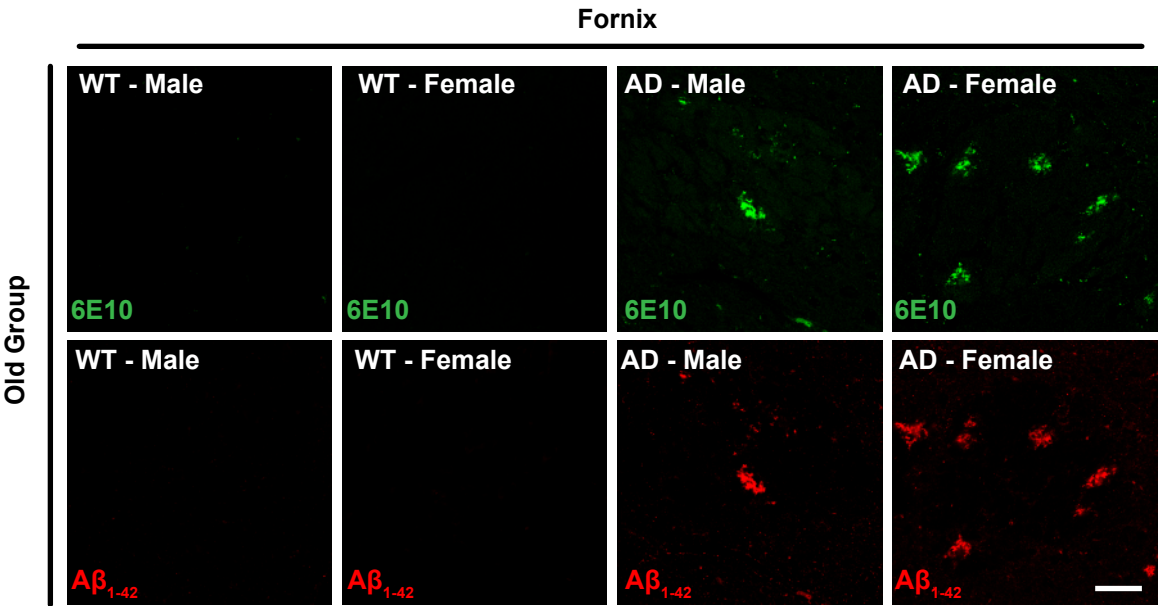

B

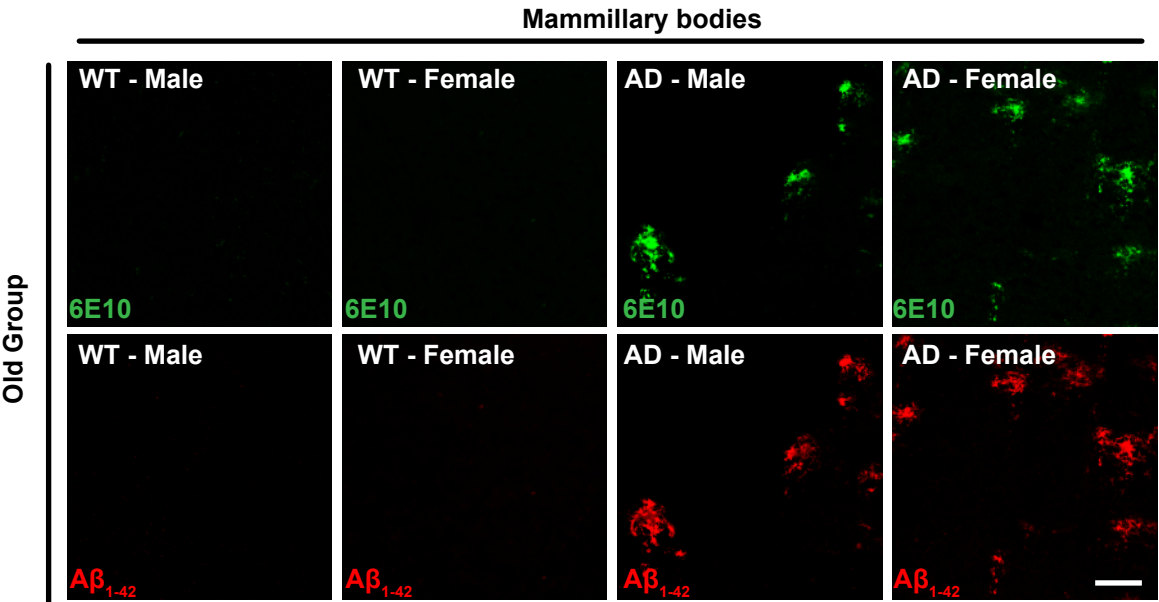

C

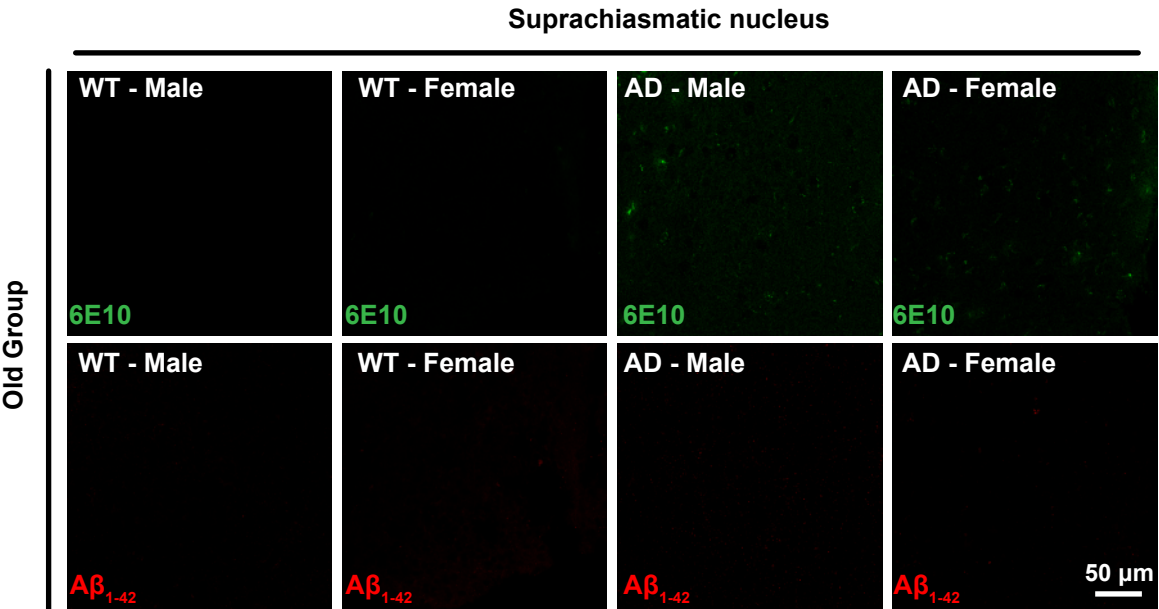

Supplement: Supplementary file 1 — Additional file 1: Fig. S1 Representative confocal images of amyloid plaques in the Old-AD-Male and Old-AD-Female subsets. 6E10+ and Aβ1–42+ plaques are observed in the fornix (A), mammillary bodies (B), but not in the suprachiasmatic nucleus. Scale bar, 50 μm. Fig. S2 Analysis of amyloid plaques in hypothalamic subdivisions of the young group. Representative images of 6E10 and Aβ1–42 co-staining in hypothalamic preoptic, anterior (A), tuberal, and mammillary (B) regions of Young-AD-Male and Young-AD-Female subsets. Fig. S3 Neurons in various hypothalamic nuclei and neuroinflammation elements. A Images of HuCD immunofluorescence in the young group. Scale bar, 200 μm. Quantification of the number of HuCD+ neurons in various nuclei. B, C Tnf-a and Ikbkβ mRNA expression in the young group were evaluated. D Images of HuCD immunofluorescence in the young group. Scale bar, 200 μm. Quantification of the number of HuCD+ neurons in various nuclei. E, F Tnf-a and Ikbkβ mRNA expression in the young group were evaluated. Fig. S4 Staining of IBA1 and GFAP in various hypothalamic nuclei. A Representative image of IBA1 staining in mammillary bodies of the young group. B Percentage of ROI occupied by IBA1-positive area (n = 3). Representative image of GFAP staining in mammillary bodies of the young group (c) and old group(d). *p < 0.05, two-way ANOVA followed by Bonferroni’s multiple comparison. Fig. S5 Vimentin-positive tanycytes distribute from zone 1 to zone 4 in the young group. Pattern of tight junction protein ZO-1 (red) is honeycombed in bottom 3V of the young group [file 13578_2024_1295_MOESM1_ESM.zip › New folder/Figure S1 plaque.pdf]

**A**

Preoptic region

Anterior region

Young Group

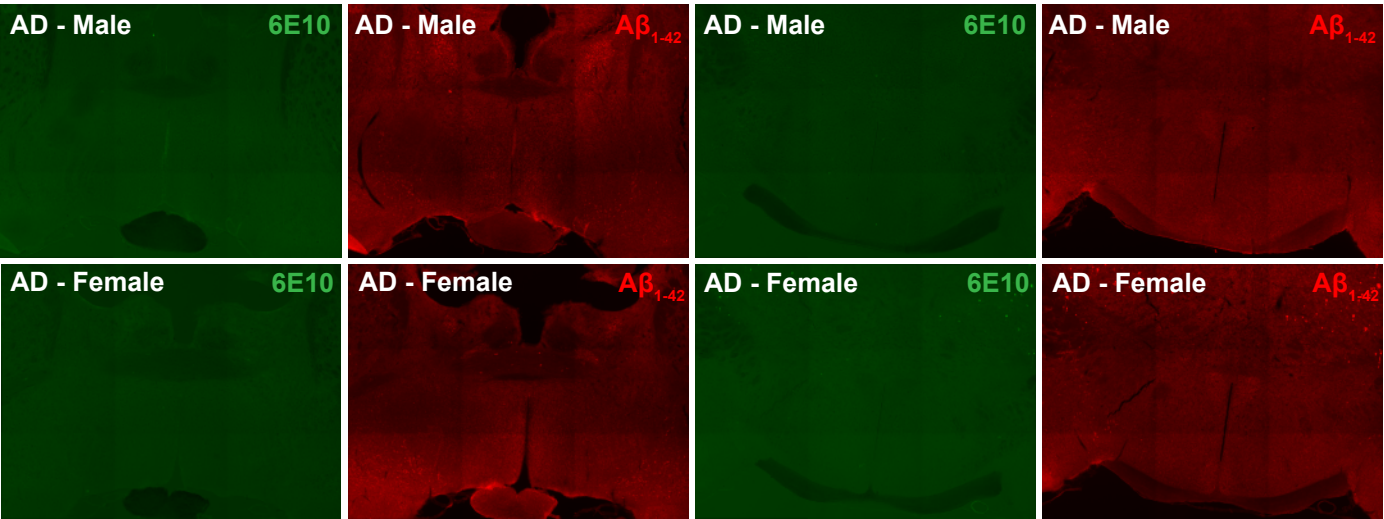

**B**

Tuberal region

Mammillary region

Young Group

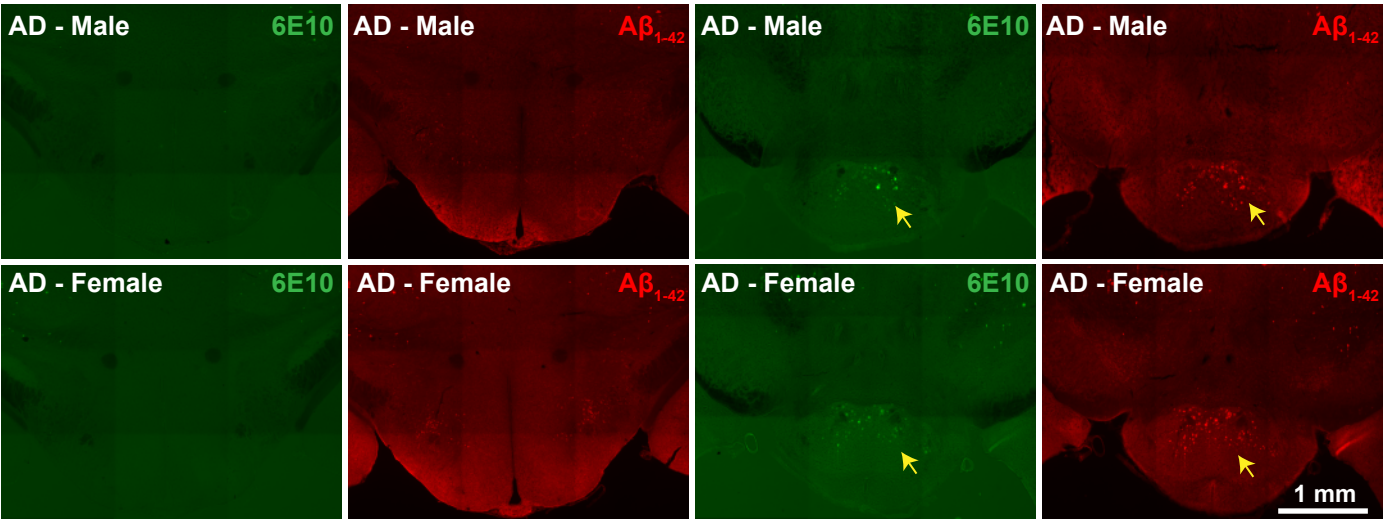

Supplement: Supplementary file 1 — Additional file 1: Fig. S1 Representative confocal images of amyloid plaques in the Old-AD-Male and Old-AD-Female subsets. 6E10+ and Aβ1–42+ plaques are observed in the fornix (A), mammillary bodies (B), but not in the suprachiasmatic nucleus. Scale bar, 50 μm. Fig. S2 Analysis of amyloid plaques in hypothalamic subdivisions of the young group. Representative images of 6E10 and Aβ1–42 co-staining in hypothalamic preoptic, anterior (A), tuberal, and mammillary (B) regions of Young-AD-Male and Young-AD-Female subsets. Fig. S3 Neurons in various hypothalamic nuclei and neuroinflammation elements. A Images of HuCD immunofluorescence in the young group. Scale bar, 200 μm. Quantification of the number of HuCD+ neurons in various nuclei. B, C Tnf-a and Ikbkβ mRNA expression in the young group were evaluated. D Images of HuCD immunofluorescence in the young group. Scale bar, 200 μm. Quantification of the number of HuCD+ neurons in various nuclei. E, F Tnf-a and Ikbkβ mRNA expression in the young group were evaluated. Fig. S4 Staining of IBA1 and GFAP in various hypothalamic nuclei. A Representative image of IBA1 staining in mammillary bodies of the young group. B Percentage of ROI occupied by IBA1-positive area (n = 3). Representative image of GFAP staining in mammillary bodies of the young group (c) and old group(d). *p < 0.05, two-way ANOVA followed by Bonferroni’s multiple comparison. Fig. S5 Vimentin-positive tanycytes distribute from zone 1 to zone 4 in the young group. Pattern of tight junction protein ZO-1 (red) is honeycombed in bottom 3V of the young group [file 13578_2024_1295_MOESM1_ESM.zip › New folder/Figure S2 plaque.pdf]

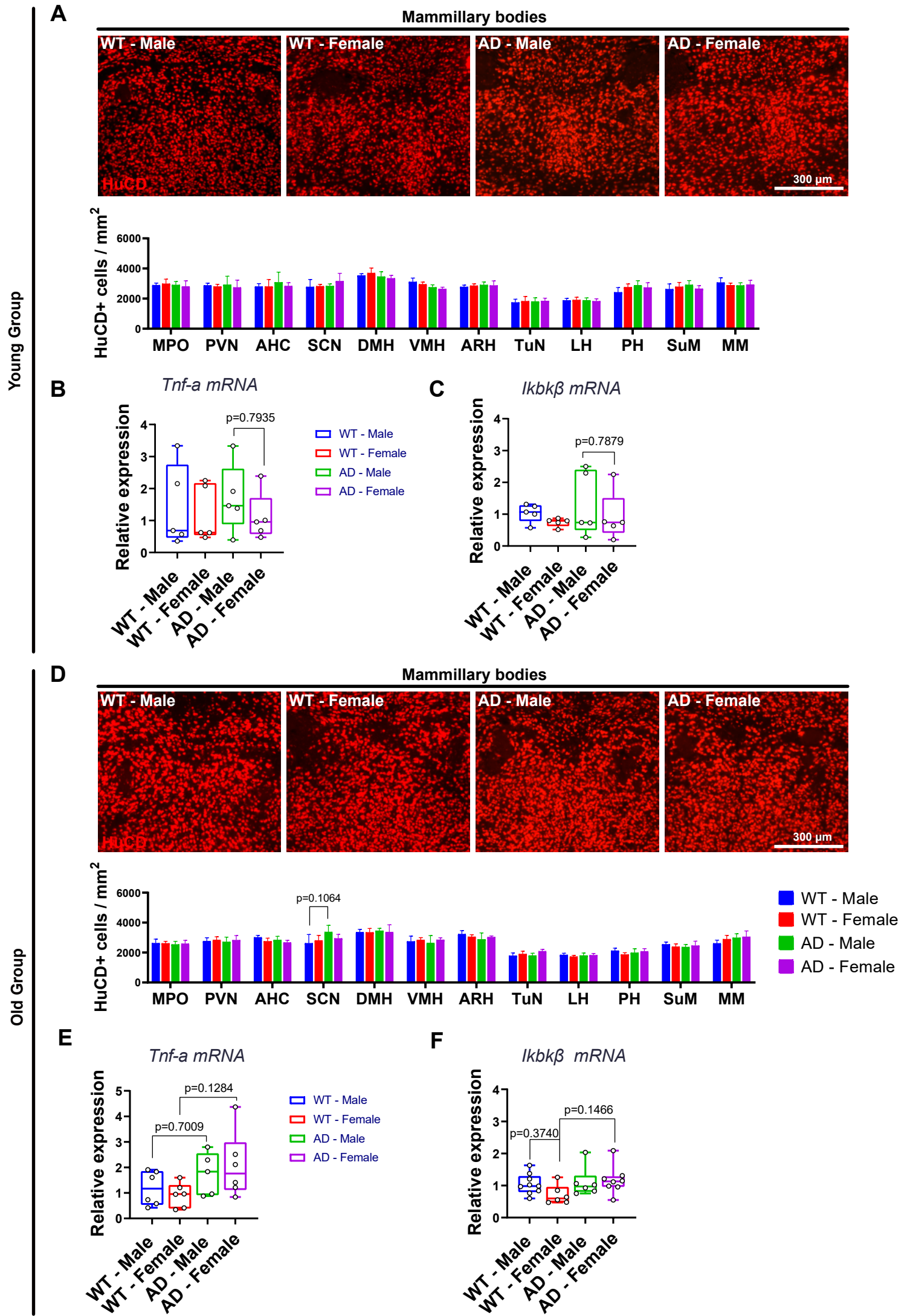

Supplement: Supplementary file 1 — Additional file 1: Fig. S1 Representative confocal images of amyloid plaques in the Old-AD-Male and Old-AD-Female subsets. 6E10+ and Aβ1–42+ plaques are observed in the fornix (A), mammillary bodies (B), but not in the suprachiasmatic nucleus. Scale bar, 50 μm. Fig. S2 Analysis of amyloid plaques in hypothalamic subdivisions of the young group. Representative images of 6E10 and Aβ1–42 co-staining in hypothalamic preoptic, anterior (A), tuberal, and mammillary (B) regions of Young-AD-Male and Young-AD-Female subsets. Fig. S3 Neurons in various hypothalamic nuclei and neuroinflammation elements. A Images of HuCD immunofluorescence in the young group. Scale bar, 200 μm. Quantification of the number of HuCD+ neurons in various nuclei. B, C Tnf-a and Ikbkβ mRNA expression in the young group were evaluated. D Images of HuCD immunofluorescence in the young group. Scale bar, 200 μm. Quantification of the number of HuCD+ neurons in various nuclei. E, F Tnf-a and Ikbkβ mRNA expression in the young group were evaluated. Fig. S4 Staining of IBA1 and GFAP in various hypothalamic nuclei. A Representative image of IBA1 staining in mammillary bodies of the young group. B Percentage of ROI occupied by IBA1-positive area (n = 3). Representative image of GFAP staining in mammillary bodies of the young group (c) and old group(d). *p < 0.05, two-way ANOVA followed by Bonferroni’s multiple comparison. Fig. S5 Vimentin-positive tanycytes distribute from zone 1 to zone 4 in the young group. Pattern of tight junction protein ZO-1 (red) is honeycombed in bottom 3V of the young group [file 13578_2024_1295_MOESM1_ESM.zip › New folder/Figure S3.pdf]

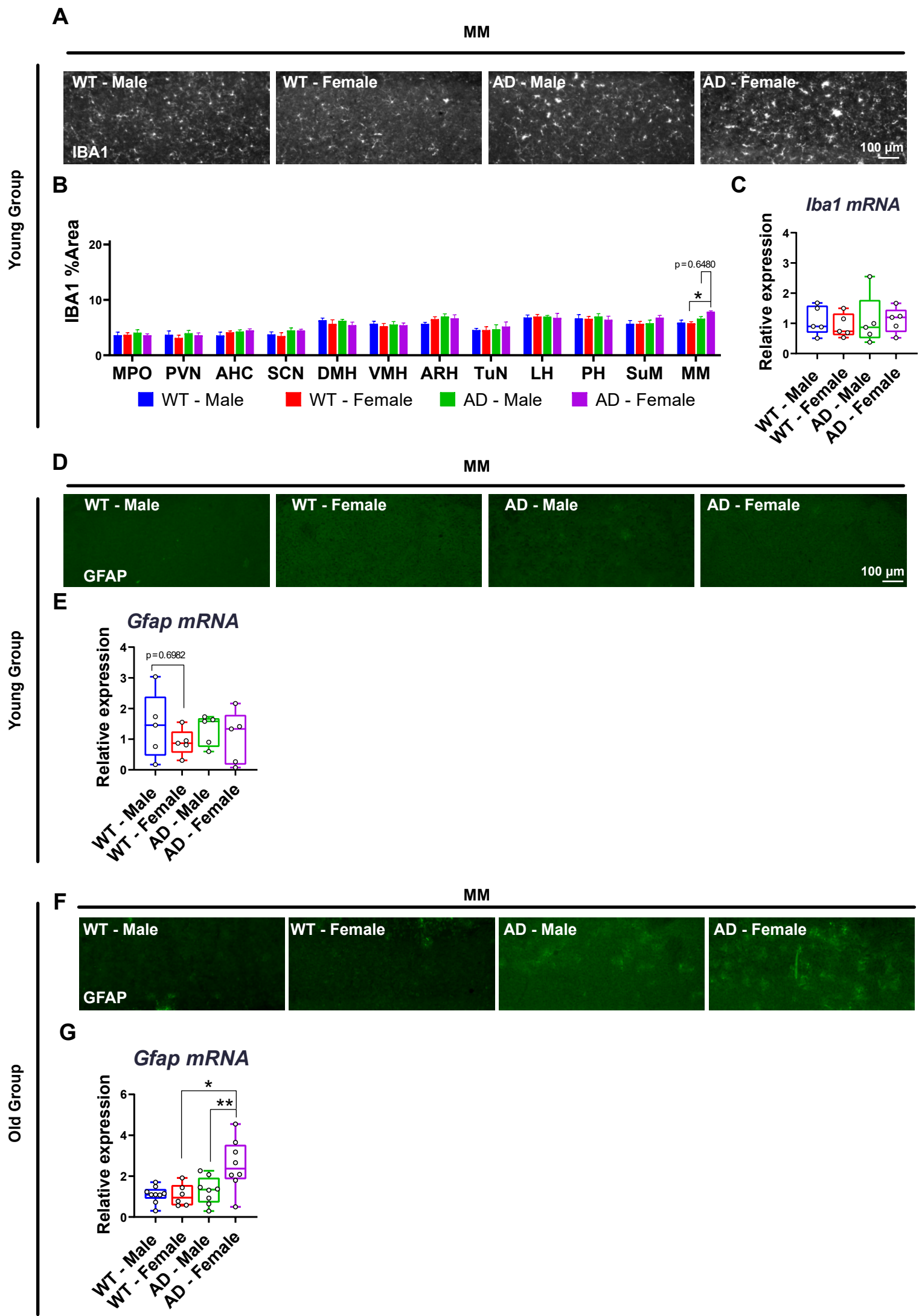

Supplement: Supplementary file 1 — Additional file 1: Fig. S1 Representative confocal images of amyloid plaques in the Old-AD-Male and Old-AD-Female subsets. 6E10+ and Aβ1–42+ plaques are observed in the fornix (A), mammillary bodies (B), but not in the suprachiasmatic nucleus. Scale bar, 50 μm. Fig. S2 Analysis of amyloid plaques in hypothalamic subdivisions of the young group. Representative images of 6E10 and Aβ1–42 co-staining in hypothalamic preoptic, anterior (A), tuberal, and mammillary (B) regions of Young-AD-Male and Young-AD-Female subsets. Fig. S3 Neurons in various hypothalamic nuclei and neuroinflammation elements. A Images of HuCD immunofluorescence in the young group. Scale bar, 200 μm. Quantification of the number of HuCD+ neurons in various nuclei. B, C Tnf-a and Ikbkβ mRNA expression in the young group were evaluated. D Images of HuCD immunofluorescence in the young group. Scale bar, 200 μm. Quantification of the number of HuCD+ neurons in various nuclei. E, F Tnf-a and Ikbkβ mRNA expression in the young group were evaluated. Fig. S4 Staining of IBA1 and GFAP in various hypothalamic nuclei. A Representative image of IBA1 staining in mammillary bodies of the young group. B Percentage of ROI occupied by IBA1-positive area (n = 3). Representative image of GFAP staining in mammillary bodies of the young group (c) and old group(d). *p < 0.05, two-way ANOVA followed by Bonferroni’s multiple comparison. Fig. S5 Vimentin-positive tanycytes distribute from zone 1 to zone 4 in the young group. Pattern of tight junction protein ZO-1 (red) is honeycombed in bottom 3V of the young group [file 13578_2024_1295_MOESM1_ESM.zip › New folder/Figure S4.pdf]

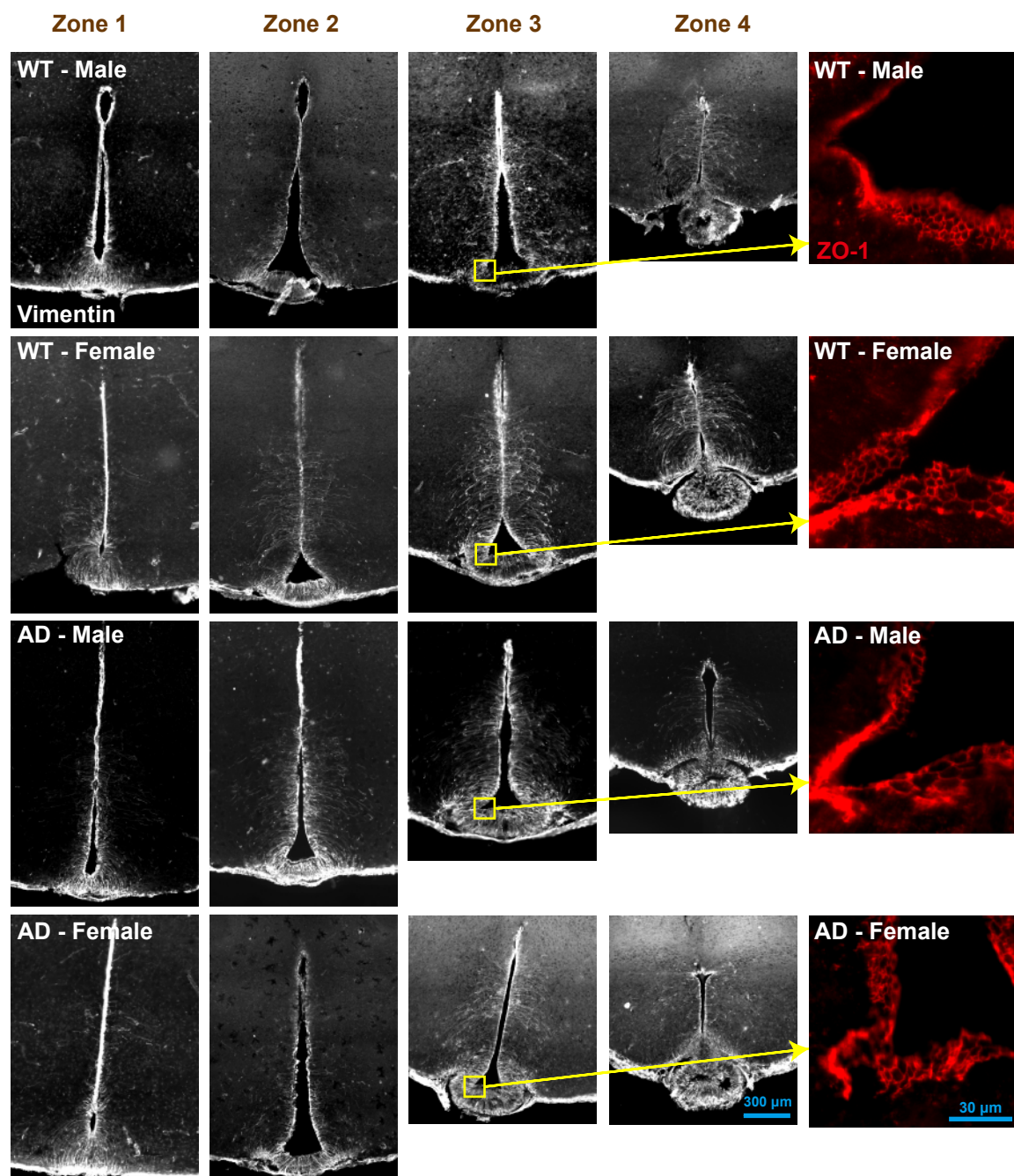

Supplement: Supplementary file 1 — Additional file 1: Fig. S1 Representative confocal images of amyloid plaques in the Old-AD-Male and Old-AD-Female subsets. 6E10+ and Aβ1–42+ plaques are observed in the fornix (A), mammillary bodies (B), but not in the suprachiasmatic nucleus. Scale bar, 50 μm. Fig. S2 Analysis of amyloid plaques in hypothalamic subdivisions of the young group. Representative images of 6E10 and Aβ1–42 co-staining in hypothalamic preoptic, anterior (A), tuberal, and mammillary (B) regions of Young-AD-Male and Young-AD-Female subsets. Fig. S3 Neurons in various hypothalamic nuclei and neuroinflammation elements. A Images of HuCD immunofluorescence in the young group. Scale bar, 200 μm. Quantification of the number of HuCD+ neurons in various nuclei. B, C Tnf-a and Ikbkβ mRNA expression in the young group were evaluated. D Images of HuCD immunofluorescence in the young group. Scale bar, 200 μm. Quantification of the number of HuCD+ neurons in various nuclei. E, F Tnf-a and Ikbkβ mRNA expression in the young group were evaluated. Fig. S4 Staining of IBA1 and GFAP in various hypothalamic nuclei. A Representative image of IBA1 staining in mammillary bodies of the young group. B Percentage of ROI occupied by IBA1-positive area (n = 3). Representative image of GFAP staining in mammillary bodies of the young group (c) and old group(d). *p < 0.05, two-way ANOVA followed by Bonferroni’s multiple comparison. Fig. S5 Vimentin-positive tanycytes distribute from zone 1 to zone 4 in the young group. Pattern of tight junction protein ZO-1 (red) is honeycombed in bottom 3V of the young group [file 13578_2024_1295_MOESM1_ESM.zip › New folder/Figure S5.pdf]
